# Learning From Past Respiratory Infections to Predict COVID-19 Outcomes: Retrospective Study

**DOI:** 10.2196/23026

**Published:** 2021-02-22

**Authors:** Shengtian Sang, Ran Sun, Jean Coquet, Harris Carmichael, Tina Seto, Tina Hernandez-Boussard

**Affiliations:** 1 Department of Medicine Biomedical Informatics Stanford University Stanford, CA United States; 2 Intermountain Health Salt Lake City, UT United States; 3 Technology and Digital Solutions Stanford University Stanford, CA United States

**Keywords:** COVID-19, invasive mechanical ventilation, all-cause mortality, machine learning, artificial intelligence, respiratory, infection, outcome, data, feasibility, framework

## Abstract

**Background:**

For the clinical care of patients with well-established diseases, randomized trials, literature, and research are supplemented with clinical judgment to understand disease prognosis and inform treatment choices. In the void created by a lack of clinical experience with COVID-19, artificial intelligence (AI) may be an important tool to bolster clinical judgment and decision making. However, a lack of clinical data restricts the design and development of such AI tools, particularly in preparation for an impending crisis or pandemic.

**Objective:**

This study aimed to develop and test the feasibility of a “patients-like-me” framework to predict the deterioration of patients with COVID-19 using a retrospective cohort of patients with similar respiratory diseases.

**Methods:**

Our framework used COVID-19–like cohorts to design and train AI models that were then validated on the COVID-19 population. The COVID-19–like cohorts included patients diagnosed with bacterial pneumonia, viral pneumonia, unspecified pneumonia, influenza, and acute respiratory distress syndrome (ARDS) at an academic medical center from 2008 to 2019. In total, 15 training cohorts were created using different combinations of the COVID-19–like cohorts with the ARDS cohort for exploratory purposes. In this study, two machine learning models were developed: one to predict invasive mechanical ventilation (IMV) within 48 hours for each hospitalized day, and one to predict all-cause mortality at the time of admission. Model performance was assessed using the area under the receiver operating characteristic curve (AUROC), sensitivity, specificity, positive predictive value, and negative predictive value. We established model interpretability by calculating SHapley Additive exPlanations (SHAP) scores to identify important features.

**Results:**

Compared to the COVID-19–like cohorts (n=16,509), the patients hospitalized with COVID-19 (n=159) were significantly younger, with a higher proportion of patients of Hispanic ethnicity, a lower proportion of patients with smoking history, and fewer patients with comorbidities (*P*<.001). Patients with COVID-19 had a lower IMV rate (15.1 versus 23.2, *P*=.02) and shorter time to IMV (2.9 versus 4.1 days, *P*<.001) compared to the COVID-19–like patients. In the COVID-19–like training data, the top models achieved excellent performance (AUROC>0.90). Validating in the COVID-19 cohort, the top-performing model for predicting IMV was the XGBoost model (AUROC=0.826) trained on the viral pneumonia cohort. Similarly, the XGBoost model trained on all 4 COVID-19–like cohorts without ARDS achieved the best performance (AUROC=0.928) in predicting mortality. Important predictors included demographic information (age), vital signs (oxygen saturation), and laboratory values (white blood cell count, cardiac troponin, albumin, etc). Our models had class imbalance, which resulted in high negative predictive values and low positive predictive values.

**Conclusions:**

We provided a feasible framework for modeling patient deterioration using existing data and AI technology to address data limitations during the onset of a novel, rapidly changing pandemic.

## Introduction

The SARS-CoV-2 virus, which causes the disease COVID-19, has infected almost 107 million people worldwide and is responsible for more than 2.3 million deaths [1]. Around the globe, patients with COVID-19 have a broad range of symptoms and disease severities. However, patients with COVID-19 demonstrate several symptoms that are striking in their commonality and nonspecificity to other well-known respiratory infections, such as pneumonia, influenza, and acute respiratory distress syndrome (ARDS) [2-5]. In fact, initial reporting of the disease indicated that most patients present with pneumonia- or influenza-like illnesses [6,7].

COVID-19 shares symptoms with other respiratory illnesses; from these diseases, we can learn about the clinical progression of patients, including the progression of patients presenting with severe hypoxemia followed by rapid deterioration, which often requires advanced life support such as invasive mechanical ventilation (IMV). Worldwide, the pandemic has left health systems struggling with capacity limits, especially regarding intensive care units (ICU) and mechanical ventilators [8,9]. Patients with COVID-19 remain on mechanical ventilation for an average of 10 to 20 days, further limiting the availability of this scarce resource [10,11]. Furthermore, an earlier study showed a high mortality rate of up to about 60% among critically ill patients [12]. The severity of cases has put great pressure on health systems, leading to a shortage of intensive care resources. Thus, understanding who will become critically ill and consume scarce resources as a result of this emerging disease can improve resource, hospital, and societal planning, and increase the number of lives saved.

However, given the novelty of COVID-19 and lack of clinical experience, the health care community is grappling for robust clinical data to learn about the disease. Under the appropriate assumptions, artificial intelligence (AI) may help with the planning of COVID-19 responses and guide clinical decisions in this time of uncertainty. Given the number of patients that may become infected with COVID-19, it is essential to understand who will be more likely to develop severe illness and need scarce resources at the time of presentation to the health care system, and AI can help with this determination. Indeed, AI technologies related to COVID-19 outcomes are emerging, as indicated in a recent published systematic review, with a majority of these studies conducted in China [13]. Predictive models using rule-based scoring tools and machine learning approaches have been applied to predict clinical deterioration in hospitalized patients and support health care providers in triaging patients when resources are limited [14-19]. However, there are many concerns and barriers to making this a reality [20], including uncertainty in the risk factors associated with disease progression, a limited number of patients whose data can be used to train and test models, and no public data sets available to test and validate models outside of a single health care setting. Of the COVID-19 AI studies published thus far, many have been designed and developed retrospectively on a small number of patient cases, limiting their validity and generalizability to other populations [15,21,22].

To help guide clinical decisions during the COVID-19 pandemic, we developed a framework to bootstrap AI models for outcomes of patients with COVID-19 using COVID-19–like cohorts to develop and train AI models to predict IMV within 48 hours and mortality using features associated with outcomes of patients with COVID-19. The COVID-19–like cohorts included patients diagnosed with bacterial pneumonia, influenza, viral pneumonia, and ARDS between 2008-2019. We tested the models’ performances on hospitalized patients with COVID-19. This framework may be particularly important in a novel and accelerated outbreak where clinicians and health care systems are forced to make difficult decisions without past experience of the specific disease at hand. In the void created by a lack of clinical experience with COVID-19, AI trained with data from COVID-19–like disorders may be an important way to bolster clinical judgment and decision making.

## Methods

### Study Design and Data Source

This retrospective study used data from electronic health records (EHRs) of patients admitted to the Stanford Healthcare Alliance (SHA) from January 1, 2008, to July 11, 2019. SHA is an integrated health system that includes an academic hospital, a community hospital, and a primary/specialty health care alliance. This study received approval from the institute’s Institutional Review Board (IRB). All source codes for this work are available at the Stanford Digital Repository [23].

### Study Cohorts

This study included two retrospective cohorts: a COVID-19–like cohort (n=16,509) and a COVID-19 cohort (n=159).

#### COVID-19–like Cohort

The COVID-19–like cohort used for model training included patients hospitalized with a diagnosis of bacterial pneumonia, influenza, viral pneumonia, unspecified pneumonia, or ARDS, using International Classification of Diseases codes (ICD-9 and ICD-10; Table S1 in Multimedia Appendix 1), between January 1, 2008, and July 11, 2019. These diseases were selected because of their similarity to COVID-19 in clinical manifestation, histological features, and disease progression [4,7,24]. Patients with complete missing lab data were excluded from the study (n=1712, 10.4%). For patients with multiple ICD codes for different conditions, the following rule was applied for disease categorization: influenza → viral pneumonia → bacterial pneumonia → unspecified pneumonia. Among these COVID-19–like cohorts, those who developed ARDS formed a separate COVID-19–like cohort.

#### COVID-19 Cohort

We included adults with a confirmed COVID-19 diagnosis who were hospitalized between March 1, 2020, and July 11, 2020. A confirmed COVID-19 diagnosis was defined as either a positive SARS-CoV-2 RNA detection test or a diagnosis code for COVID-19 (Table S1 in Multimedia Appendix 1). All patients were observed throughout their hospital encounter.

### Data Collection

Patient demographics and clinical information were captured from EHRs. We selected the most relevant features identified from the literature [2,3,5], including demographics, existing comorbid conditions, smoking history, symptoms at initial presentation, coinfection with other respiratory pathogens, and laboratory values (Textboxes 1-3). The patient's existing comorbidities, including cardiovascular disease, diabetes, cancer, hypertension, chronic respiratory disease, respiratory failure, and kidney disease, were determined over a 3-year period prior to hospital admission for the retrospective cohort. Laboratory values 2 weeks prior to and during the hospital stay were extracted for the retrospective and COVID-19 cohorts. Laboratory values on the day of IMV were excluded to ensure the values were not taken after IMV.

**Features included in the invasive mechanical ventilation and mortality model development (demographics and clinical characteristics).**

**Demographics and clinical characteristics**

- Age at admission (years)
- Gender
- Race/ethnicity
- Ever smoked (all life before admission)

**Comorbidity present (3 years before admission)**

- Cancer
- Chronic respiratory disease
- Cardiovascular disease
- Hypertension
- Type 2 diabetes
- Respiratory failure
- Kidney disease
- Alzheimer disease
- Cirrhosis

**Symptoms (15 days before admission)**

- Cough
- Dyspnea
- Tachypnea (respiratory rate >20)
- Hypoxemia (oxygen saturation ≤90%)
- Rhinorrhea
- Nose congestion
- Fever (temperature >37 C/98.6 F)
- Sputum
- Pharyngitis (sore throat)
- Headache
- Fatigue
- Conjunctivitis
- Diarrhea
- Anosmia
- Myalgias

**Features included in the invasive mechanical ventilation and mortality model development (laboratory findings).**

**Laboratory findings**

- White blood cell count, K/μL
- Lymphocyte count, K/μL
- Alanine aminotransferase, U/L
- Aspartate aminotransferase, U/L
- Creatinine, mg/dL
- Lactate dehydrogenase, U/L
- Creatine kinase, U/L
- Cardiac troponin, ng/mL
- D-dimer, ng/mL
- Prothrombin time, seconds
- Serum ferritin, ng/mL
- Procalcitonin, ng/mL
- Platelet count, K/μL
- C-reactive protein, mg/dL
- Total bilirubin, mg/dL
- Blood urea nitrogen, mg/dL
- Albumin, g/dL
- Oxygen saturation, mm Hg
- Fraction of inspired oxygen, %
- Sodium, mmol/L
- Potassium, mmol/L

**Features included in the invasive mechanical ventilation and mortality model development (coinfections).**

**Coinfection (15 days before admission)**

- Adenovirus
- Chlamydia pneumoniae
- Coronavirus
- Influenza A
- Influenza B
- Metapneumovirus
- Mycoplasma pneumonia
- Parainfluenza 1
- Parainfluenza 2
- Parainfluenza 3
- Parainfluenza 4
- Rhinovirus/enterovirus
- Respiratory syncytial virus

### Outcome

The outcome of interest was IMV in the next 48 hours and all-cause mortality. In the COVID-19–like cohort, the need for IMV in 48 hours was identified from the EHRs using the presence of IMV date and time. This time point was selected due to the rapid deterioration of disease conditions observed in patients with COVID-19. Deceased individuals who did not receive IMV during their hospital stay were considered nonintubated patients. All-cause mortality was identified using the death date recorded in the EHRs, including those who died during their hospital stay and within two weeks of hospital discharge.

### Artificial Intelligence Framework

**Figure 1:**
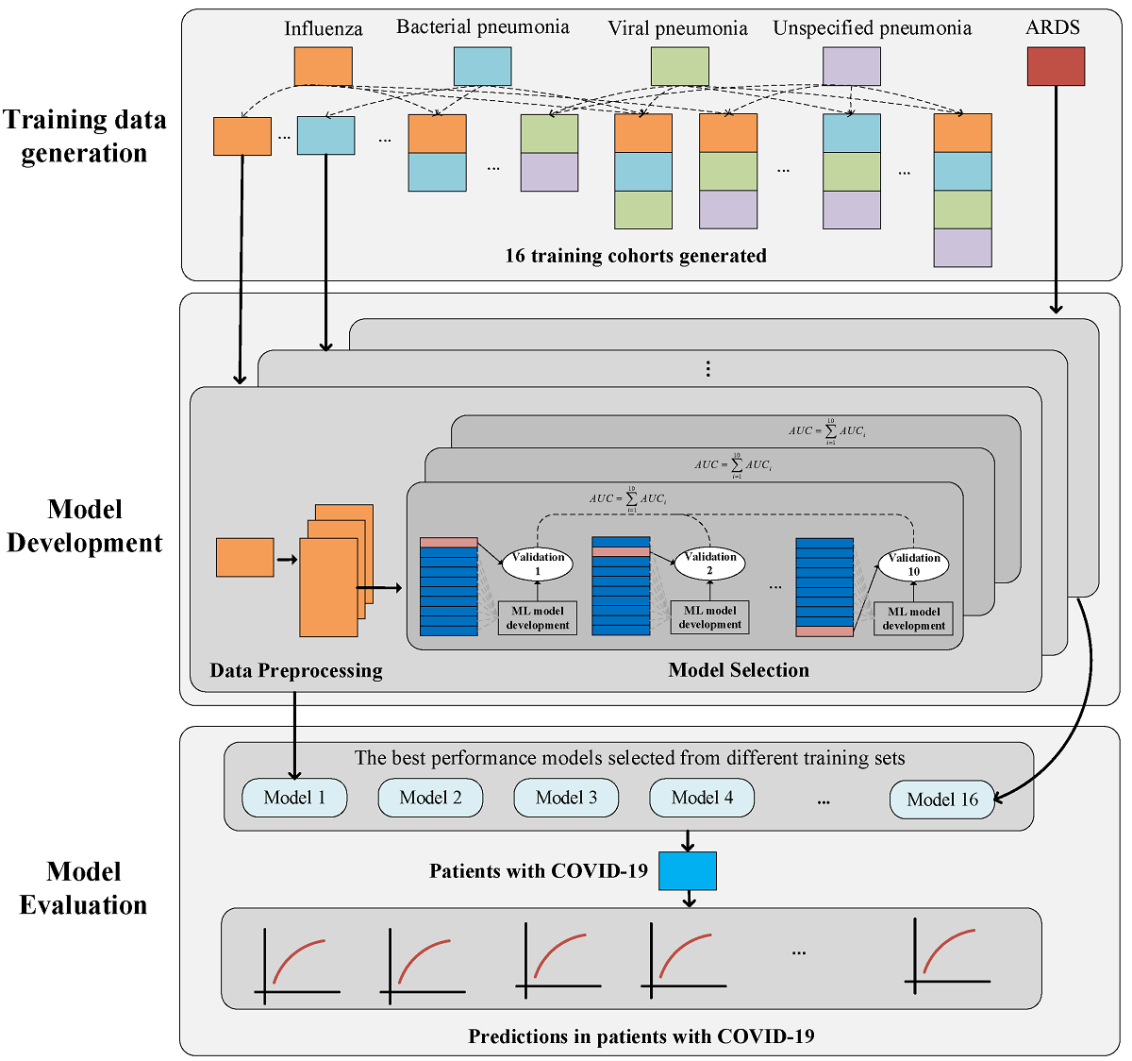
The methodological framework for selecting training data cohort, model development, and model evaluation for predicting intubation and mortality.

We developed a three-step framework to bootstrap machine learning models to predict IMV and mortality among hospitalized patients with COVID-19. Figure 1 illustrates the study design framework, including training data generation, model development, and model evaluation.

#### Training Data Generation

To determine the most appropriate COVID-19–like cohort for the prediction task, we explored different combinations of the 4 COVID-19–like cohorts (influenza, bacterial pneumonia, viral pneumonia, and unspecified pneumonia), including any single disease, any two diseases, any three diseases, and all four diseases combined to construct 15 training cohorts. Due to the differences ﻿between patients with COVID-19 and patients with ARDS, these COVID-19–like patients who developed ARDS were considered as an additional exploratory cohort to examine whether or not it could contribute to better prediction for the patients with COVID-19. Since we selected the best-performing machine learning model from each COVID-19–like cohort, 16 models were developed from the 16 COVID-19–like cohorts.

#### Model Development

##### Data Preprocessing

Features with missing values in the training set were imputed as –1. Due to the unbalanced data in training cohorts (Table S2 in Multimedia Appendix 1), oversampling approaches were used to generate synthetic samples for the minority class and to balance the positive and negative training set. These approaches included synthetic minority oversampling technique (SMOTE), borderline SMOTE, support vector machine (SVM) SMOTE, and random undersampling methods. Random undersampling was initially applied to trim the number of examples in the majority class to twice that of the minority group (positive cases), then the oversampling method was used to synthesize the minority class to balance the class distribution [25].

##### Model Training

Each of the 16 preprocessed COVID-19–like cohorts were split into 70% training and 30% validation sets with stratified random sampling. We derived four machine learning algorithms, including SVM and three tree-based ensemble algorithms (decision tree, AdaBoost, and XGBoost). We selected three decision tree–based algorithms because they have previously been applied to predict clinical events in patients with respiratory diseases based on EHR data [16,26,27]. We included models that were frequently applied for clinical prediction of severe patient outcomes [16,26,28]. In total, two steps were involved in model training: (1) using the training data set, a 10-fold cross-validation strategy was used to train the machine learning models, while grid search technique was used to search all combinations of hyperparameters and determine the best hyperparameters, and (2) using all training data, the models were retrained with the best hyperparameters (obtained in step 1). The validation data were used to monitor the performance of the model to avoid overfitting in training data. The final model was derived when the performance of the model on the validation data set did not improve after 20 training iterations. The detailed processes of model training are presented in Figure S1 in Multimedia Appendix 1.

#### Model Evaluation

##### COVID-19–like Cohorts

The performance of the models on the training cohorts was compared using the area under the receiver operating characteristic curve (AUROC). The default threshold of 0.5 was selected for interpreting probabilities to class labels. For IMV, to be a true positive, the model had to predict the need for IMV within 48 hours with a risk score of ≥0.5, and the patient had to receive IMV within this time interval. If the risk score was ≥0.5 and the patient did not receive IMV in 48 hours, the patient was treated as a false positive. The inverse strategy was applied for true negative and false negative cases. For mortality, patients who died during the hospital course or within two weeks after discharge with a risk score of ≥0.5 were considered as true positives. The inverse strategy was applied for the true negative and false negative mortality cases. To interpret the model, feature importance for predicting IMV and mortality were presented using SHapley Additive exPlanations (SHAP) values [29].

##### COVID-19 Cohort

The best training cohort was selected based on model performance (ie, AUROC) on the COVID-19 cohort. To determine the feasibility of models trained on COVID-19–like cohorts to identify patients at high risk of IMV within 48 hours and all-cause mortality, we calculated the AUROC, sensitivity, specificity, positive predictive value (PPV), and negative predictive value (NPV) for patients with COVID-19.

### Statistical Analysis

Descriptive statistics were used to compare the characteristics of the COVID-19–like and COVID-19 populations. An independent *t* test or Mann-Whitney *U* test were used wherever appropriate for comparing continuous features. The Pearson chi-square test was used for categorical features, and the Fisher exact test was used when the number in the cell was <5. Algorithms were developed using the training cohort and assessed on the independent validation cohort, which played no role in model development, by calculating the PPV and AUROC. Algorithms were further tested on the independent COVID-19 cohort. A threshold of 0.50 was set for each model, and PPV and all other threshold-dependent performance metrics were derived at this alert rate. As the PPV is threshold dependent, AUROC was also compared among models. We chose to present AUROC values because they are a threshold-independent measure of discrimination. Statistical significance for primary analysis was set at *P*<.05. All tests were two-tailed.

## Results

### Cohort Description

There were a total of 16,509 patients in the COVID-19–like cohorts, and 159 patients in the COVID-19 cohort (Table 1). Compared to the COVID-19–like cohorts with pneumonia and/or influenza, patients with COVID-19 were significantly younger (mean 56.9 [SD 20.3] years versus mean 65.8 [SD 19.3] years, *P*<.001), less often White (27% versus 58.2%, *P*<.001), and more often Hispanic (46.5% versus 13.6%, *P*<.001). In addition, patients with COVID-19 had a significantly smaller proportion of ever-smokers (29.6% versus 41.3%, *P*=.003) and fewer patients with COVID-19 had comorbidities (37.1% versus 67.1%, *P*<.001). There were significant differences in IMV rate and time to IMV between the COVID-19–like patients and patients with COVID-19; the patients with COVID-19 had a lower IMV rate (15.1% versus 23.2%, *P*=.02) and shorter time to IMV (2.9 [SD 3.9] days versus 4.1 [SD 7.3] days, *P*<.001). Additionally, we observed a significantly lower mortality rate in patients with COVID-19 compared to the COVID-19–like cohorts (*P*<.001).

**Table 1:** Comparison of patient demographics in COVID-19–like and COVID-19 cohorts^a^.

Characteristics			COVID-19–like cohort (n=16,509)													COVID-19 cohort (n=159)		*P* value^b^
			Influenza		Bacterial pneumonia		Viral pneumonia		Unspecified pneumonia		Acute respiratory distress syndrome		Total					
Total, n (%)			1076 (6.5)		2779 (16.8)		681 (4.1)		10,308 (62.4)		1665 (10.1)		16,509		159			
Age (years), mean (SD)			63.9 (20.7)		63.9 (19.5)		63.8 (19.7)		67.2 (18.9)		62.3 (18.8)		65.8 (19.3)		56.9 (20.3)		<.001	
**Gender, n (%)**																		.08
	Female	557 (51.8)		1212 (43.6)		346 (50.8)		4823 (46.8)		730 (43.8)		7668 (46.4)		85 (53.5)				
	Male	519 (48.2)		1567 (56.4)		335 (49.2)		5485 (53.2)		933 (56.0)		8839 (53.5)		74 (46.5)				
**Race, n (%)**																		<.001
	White	585 (54.4)		1674 (60.2)		376 (55.2)		6096 (59.1)		872 (52.4)		9603 (58.2)		43 (27.0)				
	Asian	178 (16.5)		429 (15.4)		123 (18.1)		1640 (15.9)		284 (17.1)		2654 (16.1)		23 (14.5)				
	Black	72 (6.7)		130 (4.7)		38 (5.6)		598 (5.8)		110 (6.6)		948 (5.7)		<10 (<6.3)				
	Other	241 (22.4)		546 (19.6)		144 (21.2)		1974 (19.2)		399 (24.0)		3304 (20.0)		76 (47.8)				
**Ethnicity, n (%)**																		<.001
	Non-Hispanic	898 (83.5)		2321 (83.5)		568 (83.4)		8786 (85.2)		1335 (80.2)		13,908 (84.2)		81 (50.9)				
	Hispanic	170 (15.8)		380 (13.7)		106 (15.6)		1325 (12.9)		267 (16.0)		2248 (13.6)		74 (46.5)				
**Insurance, n (%)**																		<.001
	Private	263 (24.4)		605 (21.8)		176 (25.8)		2160 (21.0)		414 (24.9)		3618 (21.9)		32 (20.1)				
	Public	747 (69.4)		1974 (71.0)		477 (70.0)		7447 (72.5)		1129 (67.8)		11,804 (71.5)		101 (63.5)				
	Other	66 (6.2)		200 (7.2)		28 (4.1)		671 (6.5)		122 (7.4)		1087 (6.6)		26 (16.4)				
Ever smoked, n (%)			402 (37.4)		1128 (40.6)		283 (41.6)		4375 (42.4)		635 (38.1)		6823 (41.3)		47 (29.6)		.003	
At least one comorbidity^c^			771 (71.7)		1745 (62.8)		571 (83.8)		7000 (67.9)		997 (59.9)		11,084 (67.1)		59 (37.1)		<.001	
Invasive mechanical ventilation, n (%)			91 (8.5)		1031 (37.1)		95 (14.0)		1811 (17.6)		797 (47.9)		3825 (23.2)		24 (15.1)		.02	
Time to invasive mechanical ventilation (days), mean (SD)			4.4 (7.2)		3.8 (6.7)		6.9 (12.9)		4.0 (7.2)		4.3 (7.4)		4.1 (7.3)		2.9 (3.9)		<.001	
All-cause mortality, n (%)			62 (5.8)		513 (18.5)		82 (12.0)		1586 (15.4)		541 (32.5)		2789 (16.9)		<10 (<6.3)		<.001	

^a^Percentages may not add up to 100 due to missing values.

^b^The *P* value was calculated between the total of 4 retrospective COVID-19–like cohorts and the COVID-19 cohort.

^c^Comorbidities include cardiovascular disease, diabetes, cancer, hypertension, chronic respiratory disease, respiratory failure, kidney disease, Alzheimer disease, and cirrhosis.

Tables 2 and 3 compare symptoms at presentation and laboratory results during a hospital course between the COVID-19–like cohort and patients with COVID-19. Patients with COVID-19 experience a wider range and greater number of symptoms compared to the COVID-19–like cohort with pneumonia and influenza (*P*<.05). The average clinical laboratory results during hospitalization were also different (Table 3). The median laboratory values of D-dimer, lactate dehydrogenase, and ferritin elevated in hospitalized patients with COVID-19. Compared to the retrospective cohort of hospitalized patients with pneumonia and influenza, patients with COVID-19 had significantly higher values for lymphocyte count, platelet count, aspartate aminotransferase, albumin, lactate dehydrogenase, and ferritin, and lower values for white blood cell count, bilirubin, blood urea nitrogen, D-dimer, procalcitonin, creatinine, and prothrombin time.

**Table 2:** Comparison of symptoms at admission of the COVID-19–like cohort and patients with COVID-19.

Symptoms at admission	COVID-19–like cohort (n=16,509), n (%)	COVID-19 cohort (n=159), n (%)	*P* value
Cough	2643 (16.0)	116 (73.0)	<.001
Dyspnea	6087 (36.9)	119 (74.8.)	<.001
Fever	3147 (19.1)	103 (64.8)	<.001
Fatigue	2046 (12.4)	67 (42.1)	<.001
Myalgias	243 (1.5)	52 (32.7)	<.001
Hypoxemia	3910 (23.7)	51 (32.1)	.01
Headache	638 (3.9)	28 (17.6)	<.001
Diarrhea	1784 (10.8)	29 (18.2)	.003
Tachypnea	821 (5.0)	57 (35.8)	<.001
Pharyngitis (sore throat)	53 (0.3)	28 (17.6)	<.001
Sputum	0 (0.0)	26 (16.4)	N/A
Rhinorrhea	0 (0.0)	6 (3.8)	N/A
Nasal congestion	62 (0.4)	10 (6.3)	<.001
Anosmia	8 (0.0)	20 (12.6)	N/A

**Table 3:** Comparison of laboratory values of the COVID-19–like cohort and patients with COVID-19.

Laboratory values	Values missing, n (%)	Median (IQR)	Values missing, n (%)	Median (IQR)	*P* value
White blood cell count, K/μL	2767 (16.8)	8.9 (5.7)	0 (0.0)	6.8 (3.5)	<.001
Lymphocyte count, K/μL	3386 (20.5)	1.0 (0.8)	2 (1.3)	1.1 (0.7)	.008
Platelet count, K/μL	2768 (16.8)	197.3 (134.5)	0 (0.0)	240.9 (110.7)	<.001
Alanine aminotransferase, units/L	3977 (24.1)	32.0 (25.5)	1 (0.6)	33.2 (39.7)	.65
Aspartate aminotransferase, units/L	3803 (23.0)	29.0 (24.9)	1 (0.6)	40.9 (28.6)	<.001
Total bilirubin, mg/dL	5125 (31.0)	0.6 (0.5)	2 (1.3)	0.4 (0.3)	<.001
Albumin, g/dL	3744 (22.7)	2.8 (1.0)	1 (0.6)	3.4 (0.5)	<.001
Blood urea nitrogen, mg/dL	2818 (17.1)	19.0 (16.5)	0 (0.0)	13.5 (10.3)	<.001
Troponin I, ng/mL	12,026 (72.8)	0.06 (0.18)	135 (84.9)	0.05 (0.14)	.82
D-dimer, ng/mL	14,717 (89.1)	2311.8 (2646.5)	141 (88.7)	1125.5 (976)	.001
Lactate dehydrogenase, units/L	13,793 (83.5)	293.0 (259.8)	56 (35.2)	369.0 (188.0)	<.001
Ferritin, ng/mL	14,763 (89.4)	355.0 (819.2)	55 (34.6)	788.5 (875.8)	<.001
Procalcitonin, ng/mL	13,965 (84.6)	0.5 (1.4)	52 (32.7)	0.1 (0.2)	<.001
C-reactive protein, mg/dL	15,225 (92.2)	8.7 (13.7)	71 (44.7)	7.2 (10.2)	.30
Creatine kinase, units/L	13,668 (82.8)	76.0 (143.5)	93 (58.5)	81.5 (161.1)	.61
Sodium, mmol/L	2751 (16.7)	136.9 (4.8)	0 (0.0)	137.0 (4.3)	.56
Potassium, mmol/L	2750 (16.7)	4.0 (0.5)	0 (0.0)	4.0 (0.5)	.97
Creatinine, mg/dL	2757 (16.7)	1.0 (0.6)	0 (0.0)	0.8 (0.3)	<.001
Prothrombin time, s	6322 (38.3)	15.2 (3.2)	43 (27.0)	13.6 (1.5)	<.001
Oxygen saturation, %	7107 (43.0)	96.0 (2.5)	0 (0.0)	95.9 (2.4)	.10
Fraction of inspired oxygen, %	8125 (49.2)	28.5 (10.0)	35 (22.0)	27.7 (13.4)	.44

### Model Performance: Predicting 48-Hour IMV

**Figure 2:**
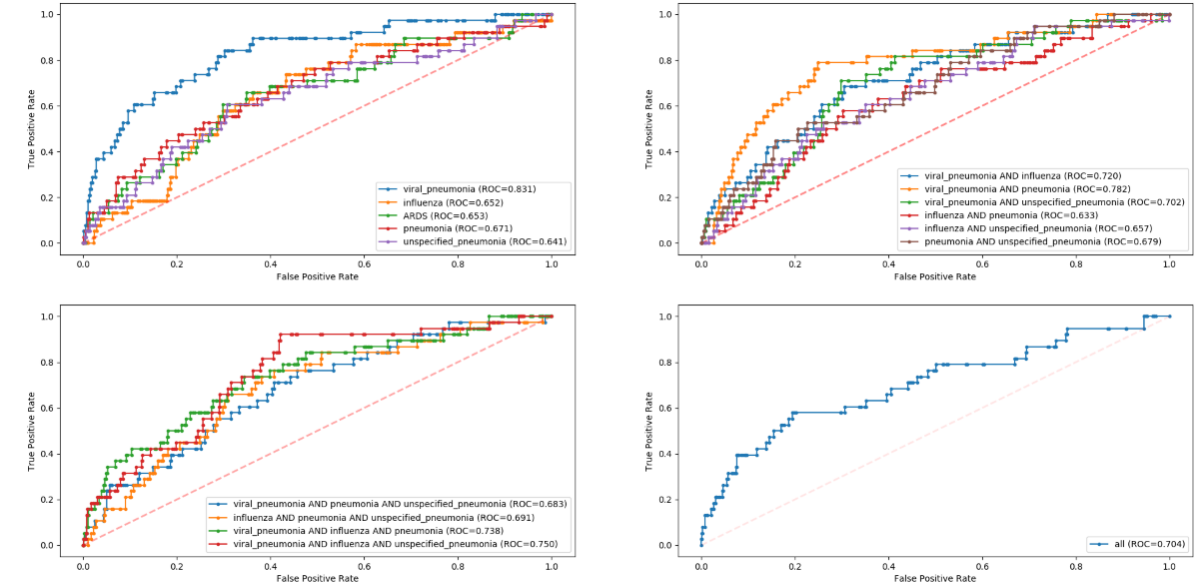
Receiver operating characteristic curves for model performance on predicting 48-hour invasive mechanical ventilation risk in the COVID-19 cohort, stratified by COVID-19–like cohorts. ARDS: acute respiratory distress syndrome; ROC: receiver operating characteristic.

The AUROCs of all models for predicting 48-hour IMV risk are illustrated in Figure 2. Algorithm discrimination and other performance metrics for the COVID-19–like cohorts are presented for each model in Table S3 in Multimedia Appendix 1. At the prespecified threshold of 0.5, the XGBoost classifiers achieved the highest AUROCs (range 0.772-0.905) compared to other machine learning classifiers in each of the 16 training cohorts for prediction of 48-hour risk for IMV. The best PPVs ranged between 0.583 and 0.767, and all models had an accuracy of 0.724 or higher and specificity of 0.786 or higher. The model trained with the influenza cohort was one of the worst-performing models, with an AUROC of 0.772 and PPV of 0.583 for IMV.

The pretrained models for 48-hour IMV prediction performed worse on the COVID-19 cohort than on the COVID-19–like cohorts. The model with the best performance on the new COVID-19 cohort was the XGBoost model trained on the viral pneumonia cohort (AUROC=0.826). The accuracy, precision, and sensitivity of the best model were 0.948, 0.423, and 0.289, respectively. Performance metrics for each model are presented in Table S4 in Multimedia Appendix 1. For negative cases at the patient-day level, the mean risk score was 0.09 (SD 0.124), with a minimum of 0.002 and a maximum of 0.859. For positive cases, the mean risk score was 0.31 (SD 0.23), with a minimum of 0.012 and a maximum of 0.823. At the patient level, the best-performing model was able to predict IMV in 48 hours for 7 of the 24 intubated patients with COVID-19. The model failed to predict the need for IMV in 48 hours for 17 patients (71%). Among the non-IMV patients, two were predicted to be at high risk of requiring IMV, although they were never intubated during hospitalization. Further details of the model hyperparameter optimization for predicting IMV can be found in Tables S5 and S6 in Multimedia Appendix 1.

### Model Performance: Predicting All-Cause Mortality

**Figure 3:**
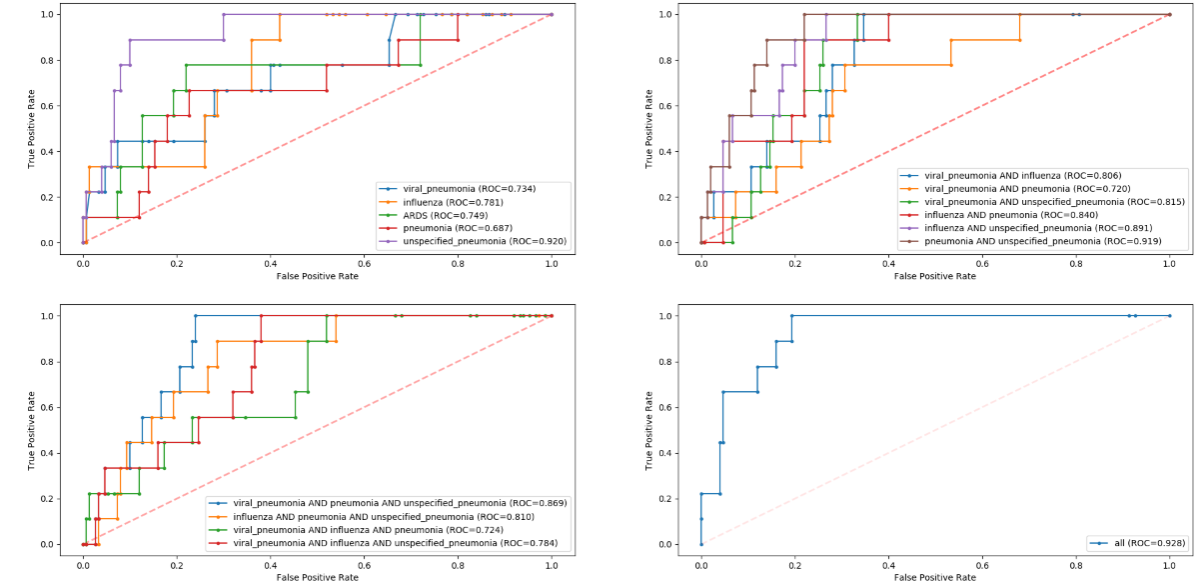
Receiver operating characteristic curves for model performance on predicting inpatient mortality in the COVID-19 cohort, stratified by COVID-19–like cohorts. ARDS: acute respiratory distress syndrome; ROC: receiver operating characteristic.

Figure 3 and Table S7 in Multimedia Appendix 1 present detailed results of model performance for mortality prediction on the COVID-19–like cohorts. The XGBoost classifiers outperformed other machine learning classifiers in each of the 16 training cohorts for all-cause mortality prediction, with AUROC values ranging from 0.679 to 0.748.

Mortality prediction was better when testing on the COVID-19 cohort, with AUROC values ranging from 0.687 to 0.928. The best-performing model for the COVID-19 cohort was the XGBoost model trained on all 4 COVID-19–like cohorts (AUROC=0.928). The accuracy, precision, and sensitivity of the best model were 0.925, 0.286, and 0.222, respectively. The worst-performing model for the COVID-19 cohort was trained by using the pneumonia cohort, and achieved an AUROC of 0.687. Other results are presented in Table S8 in Multimedia Appendix 1. For all-cause mortality prediction at the patient level, the best-performing model predicted 22% of the deaths at the time of hospital admission. Further, our model predicted 5 deaths among the 109 patients discharged alive at the time of hospital admission.

### Manual Chart Review

Manual chart review of 24 false positive and false negative cases was performed by author HC. Among the seven false positive cases for IMV prediction, one patient received IMV; however, this was not entered as structured data in the EHRs, therefore it was marked as a false positive for the model. Furthermore, two patients were extremely ill and close to receiving IMV. In the 17 false negative cases, 6 patients experienced rapid clinical deterioration in less than 24 hours or sometimes within 12 hours, and another 6 patients received scores that were close to the threshold. Additionally, our model incorrectly predicted the risk of death in 12 patients, including 5 false positive and 7 false negative cases. Based on the chart review, we confirmed that 3 of the 5 false positives were true false positives, while for the other two, the patients’ conditions were severe and they were identified by the clinician as having a higher risk of death.

### Algorithm Variable Importance

**Figure 4:**
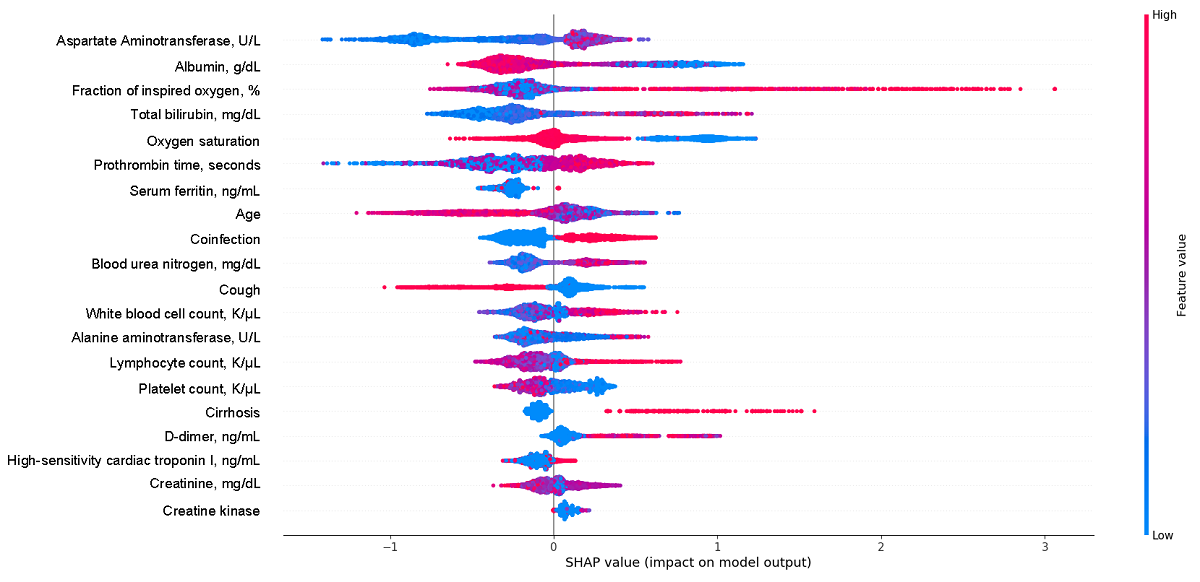
SHapley Additive exPlanations (SHAP) scores for identifying important features for prediction of invasive mechanical ventilation, including demographic information, vital values, and laboratory values. The color indicates whether the value of the feature is high (red) or low (blue).

**Figure 5:**
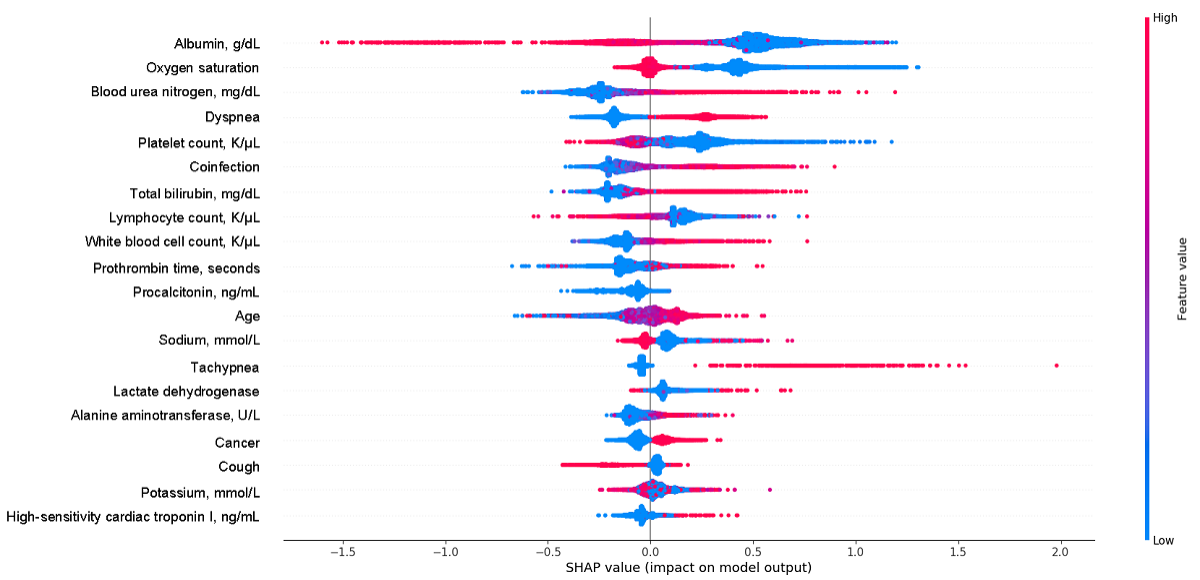
SHapley Additive exPlanations (SHAP) scores of the mortality prediction model. The color indicates whether the value of the feature is high (red) or low (blue).

To identify the most salient features driving model prediction, we calculated SHA*P* values for the best-performing model. Among the top features, elevated fraction of inspired oxygen, total bilirubin, white blood cell count, lymphocyte count, D-dimer, and cardiac troponin, and lower albumin, oxygen saturation, and platelet count favored the classifier to predict an IMV event. Other important features from the best prediction model are in Figure 4. For mortality, being older and having higher blood urea nitrogen, potassium, and high-sensitivity cardiac troponin, and low albumin, oxygen saturation, and platelet count were the most influential factors in driving mortality prediction. Other important features associated with increased mortality risk included elevated total bilirubin and lower platelet count (Figure 5).

## Discussion

### Principal Findings

In the clinical care of well-established diseases, literature and research are supplemented by the clinical judgment that is formed and refined through repeated episodes of care. Given the novelty of COVID-19, there is a lack of research evidence and clinical experience to inform clinical practice and guide care decisions. AI-enabled clinical decision support tools are promising to fill this gap and bolster clinical decision making. Early studies suggest that the disease manifestation, symptoms, and clinical course of COVID-19 resemble that of other respiratory infections, particularly pneumonia, influenza, and ARDS [5,30-32]. Due to the lack of robust, unbiased, representative data to train an AI model, we designed a framework to bootstrap existing retrospective data from COVID-19–like cohorts to predict IMV and all-cause mortality.

Our findings regarding the study cohort are consistent with previous international studies comparing patients with COVID-19 with patients previously hospitalized with other respiratory illnesses. The patients hospitalized with COVID-19 in our health system were younger and had fewer comorbidities than the COVID-19–like patients [33]. Patients with COVID-19 were less likely to have ever smoked compared to the COVID-19–like cohort. In addition, racial and ethnic minorities have been disproportionately affected by the disease, and we show similar trends in our health care system [32,34]. In addition to the variations in demographics and clinical outcomes, we observed differences in symptoms at admission and laboratory values during the hospital stay. Importantly, significant differences were observed in IMV rates, time to IMV, and mortality rates between the two cohorts, with lower IMV and mortality rates in the patients with COVID-19 than in the COVID-19–like cohort, yet faster deterioration, as indicated by a shorter duration from admission to IMV.

The models, in general, performed well in the COVID-19–like cohorts, but less optimally in the COVID-19 cohort. Although the AUROC values of the best IMV and mortality prediction models in the COVID-19 cohort were good, the PPVs of both models were low, although the NPVs were high. Overall, the two models underestimated risk scores in patients with COVID-19. This can be explained by several factors. First, symptoms, laboratory values, and the proportions of missing data were different between COVID-19–like patients and patients with COVID-19, despite them sharing similar clinical manifestations and symptoms. Second, unlike the COVID-19–like cohorts, patients with COVID-19 had a broad spectrum of clinical manifestations, with critical courses that may involve fast deterioration and the need for IMV within 24 hours; thus, patients may have had limited signs of severe disease progression 48 hours prior to IMV. On the other hand, several clinical circumstances may affect clinical decisions. Clinicians may be reluctant to put patients on a ventilator due to the complexity of and complications associated with this invasive procedure. Patients or family members might also be hesitant to consent to the procedure out of fear of losing control [35]. Sometimes, patients have a Do Not Intubate code status, indicating they do not want to receive IMV in the event of a life-threatening situation. All these factors challenge the performance of our model, particularly the PPV. The model made a positive prediction for patients who require IMV, yet patients may not receive it in the end due to the factors mentioned above, and these are therefore considered false positives.

Traditional machine learning evaluation criteria, such as AUROC and PPV, were used to assess the performance of predicting the risk of IMV and all-cause mortality at the patient-day level; however, there is a lack of standard criteria to evaluate the model at the patient level when there are also multiple day-level data. When reporting the patient-level prediction results, a “strict” criterion was selected: the model correctly predicted the case only when the alarm occurred at 48 hours before IMV. If the prediction occurred too early or too close to IMV, it was considered wrong for that patient. If correct cases were determined as at least one alarm before IMV for those who were finally intubated and no alarm for non-IMV patients, the model performance would be greatly improved. The use of different standards for analyzing patient-level results can have clinical significance. For example, if a patient is predicted to be intubated during hospitalization, the provider needs to be reminded to pay more attention to avoid the rapid deterioration of the patient’s condition. Therefore, our framework provides important insight into the deterioration of patients with COVID-19 and the timing of that deterioration. Further studies are needed to explore evaluation criteria for this novel, emerging disease.

The performances of our COVID-19–like models suggest that the deterioration in the COVID-19 population in our health system is more similar to viral pneumonia than other respiratory illnesses. These results support our evolving understanding of the clinical characteristics of this disease state and support evidence that patients with COVID-19 are less like ARDS patients than was originally believed [36]. Although the scientific community has been shifting practices of treating the ventilation needs of patients with COVID-19 away from mimicking ARDS treatment, this work may be the first indication that there is a detectable demographic and pathophysiologic difference in the presenting characteristics of COVID-19 as well as the response to therapy.

This work has several clinical applications. It is notable that this model could make for an excellent screening tool for clinical deterioration in the inpatient setting because the model has a high positive likelihood ratio and high specificity. This indicates that a positive result truly indicates an increased probability of clinical respiratory decline for those admitted to hospital with COVID-19, even if the individual PPV for that “patient-day” is low. The implementation of this model would allow for enhanced monitoring of patients likely to require advanced respiratory support, especially during surge settings when there is a strain on staffing with advanced infectious disease or pulmonary training.

Although there is lower ability to predict patients in need of IMV within 48 hours or all-cause mortality at the “patient-day” level with great precision, this work can also be used to identify patients who are not at risk of clinical escalation. This means that this model can be used as a screening tool in our population, offering providers some confidence in the current level of care being appropriate rather than using valuable hospital resources on enhanced monitoring for patients who are less likely to need advanced medical management for respiratory failure. Such information can help with resource allocation and help providers triage patients who are less likely to become critically ill. Given the extreme stress and burden the COVID-19 pandemic has placed on the health care system, particularly on frontline workers, identifying patients who may need less focused attention may reduce some of the burden for health care systems that are already stretched thin. We anticipate that this work could be implemented across health care systems and therefore provide all codes and software needed to deploy these models. The external validation of our framework across systems could help in elucidating the clinical course of COVID-19 by refining the model in populations over time.

### Limitations

While we envision many possible applications of our framework, we also recognize several limitations. First, our data come from a single health care system, and the results may not generalize to other health care systems that may have a different patient population or clinical practice. External validation would be required to reinforce our conclusions. Second, the sample sizes of the COVID-19–like and COVID-19 cohorts are different, which may bias our comparison in terms of demographics and clinical characteristics. However, despite this difference, these patients share similar clinical manifestations, histological features, and disease progression. Third, our data set contained a relatively small number of deaths, and the model performance on the COVID-19 cohort could be unstable based on the limited number of patients. Future work is needed to validate the mortality prediction in other settings. Finally, the prediction of the risk of mortality used data from admission, limiting the performance of the model. Despite these limitations, important lessons have been learned from our experience of using pretrained machine learning models for disease severity prediction. It is feasible to pretrain a model using an unseen disease-like cohort but this requires special caution. First, selecting the most appropriate cohort, one which is similar to the clinical manifestation, pathological features, and disease progression of the targeted disease population, is essential for developing a successful machine learning model. Second, based on the nature of the disease and the type of data that are available, determining the right time frame for your machine learning model is crucial. We failed to predict whether or not a patient with COVID-19 would need IMV during their hospital stay using data obtained at hospital admission. Predictions may be hampered by the rapid deterioration seen in some patients with COVID-19 and changes in laboratory results.

### Conclusions

In conclusion, our work demonstrates the feasibility of using existing data infrastructure and AI technology to guide critical care resource allocation in the early stages of a disease outbreak when not many cases have been observed and there is a lack of training data. Although the spread of COVID-19 has been exponential worldwide, most individual health care systems do not have a comprehensive, diverse, readily available data set of patients with COVID-19, which is necessary to develop, train, and validate essential AI models that may be used to guide clinical care. To date, many COVID-19–related AI models distributed through the scientific community have been trained and “validated” on only a handful of patients. However, given the lack of knowledge related to COVID-19, there is an urgent need to learn as much as possible about the disease, even if from small nonrepresentative populations. The framework we describe provides a strategy to mitigate this lack of data by identifying how and what we can learn from other COVID-19–like diseases. As we will likely deal with another wave of COVID-19 cases and other pandemics in the future, having a framework to rapidly design and train predictive models will have eminent value. Although using these COVID-19–like cohorts to learn about and predict outcomes of patients with COVID-19 may not be ideal, they provide an unbiased pathway to help guide clinical decisions when faced with this novel disease.

## Appendix

Multimedia Appendix 1 Supplementary materials.

## Abbreviations

AI: artificial intelligence
ARDS: acute respiratory distress syndrome
AUROC: area under the receiver operating characteristic curve
IMV: invasive mechanical ventilation
NPV: negative predictive value
PPV: positive predictive value
SMOTE: synthetic minority oversampling technique
SVM: support vector machine
