# Supplementary material for: Learning From Past Respiratory Infections to Predict COVID-19 Outcomes: Retrospective Study
Source: J Med Internet Res. 2021 Feb 22;23(2):e23026. doi: 10.2196/23026 (PMC7901593; doi:10.2196/23026)

Multimedia Appendix 1

Table S1. ICD-9 and ICD-10 diagnosis codes for COVID-like and COVID-19 cohorts.

| Disease | ICD-9 | ICD-10 |
| --- | --- | --- |
|  |  |  |
| **COVID-Like Cohort** |  |  |
| viral pneumonia | 480.x | J12.x |
| pneumonia | 481  482.x  483.x | J13  J14  J15.x  J16.x |
| influenza | 487.x  488.x | J09.x  J10.0x  J10.1  J11.0x  J11.1 |
| unspecified pneumonia | 485  486 | J18.x |
| ARDS | 518.82 | J80 |
| **COVID-19 Cohort** |  |  |
| COVID-19 | N/A | U07.1  J12.89, B97.29  J22, B97.29  J80, B97.29 |

Table S2. Number of training cases by disease in the retrospective cohort.

| Cohort | Positive patient days | Negative patient days |
| --- | --- | --- |
|  |  |  |
| ARDS | 815 | 9,335 |
| Pneumonia | 944 | 17,112 |
| Influenza | 93 | 5,929 |
| Viral pneumonia | 116 | 4,882 |
| Unspecified pneumonia | 1,828 | 67,491 |

Table S3. Performance of different machine learning models for IMV prediction on COVID-like cohorts.

| Cohort | AUC | ACC^a^ | PPV^b^ | Sensitivity | F-score | Specificity | NPV^c^ | PLR^d^ |
| --- | --- | --- | --- | --- | --- | --- | --- | --- |
|  |  |  |  |  |  |  |  |  |
| influenza AND pneumonia | 0.905 | 0.829 | 0.758 | 0.714 | 0.735 | 0.886 | 0.861 | 6.263 |
| viral pneumonia | 0.901 | 0.750 | 0.667 | 0.500 | 0.571 | 0.875 | 0.778 | 4.000 |
| unspecified pneumonia | 0.897 | 0.830 | 0.756 | 0.723 | 0.739 | 0.883 | 0.864 | 6.179 |
| pneumonia AND unspecified pneumonia | 0.896 | 0.825 | 0.738 | 0.736 | 0.737 | 0.870 | 0.868 | 5.662 |
| viral pneumonia AND unspecified pneumonia | 0.891 | 0.830 | 0.767 | 0.705 | 0.735 | 0.893 | 0.858 | 6.589 |
| viral pneumonia AND pneumonia AND unspecified pneumonia | 0.891 | 0.825 | 0.749 | 0.714 | 0.731 | 0.881 | 0.860 | 6.000 |
| influenza AND pneumonia AND unspecified pneumonia | 0.890 | 0.818 | 0.742 | 0.698 | 0.719 | 0.878 | 0.853 | 5.721 |
| viral pneumonia AND influenza AND unspecified pneumonia | 0.889 | 0.820 | 0.733 | 0.723 | 0.728 | 0.868 | 0.863 | 5.477 |
| influenza AND unspecified pneumonia | 0.887 | 0.821 | 0.735 | 0.724 | 0.730 | 0.870 | 0.863 | 5.569 |
| pneumonia | 0.886 | 0.819 | 0.728 | 0.728 | 0.728 | 0.864 | 0.864 | 5.353 |
| viral pneumonia AND pneumonia | 0.879 | 0.821 | 0.743 | 0.708 | 0.725 | 0.877 | 0.857 | 5.756 |
| all | 0.879 | 0.810 | 0.720 | 0.704 | 0.712 | 0.863 | 0.854 | 5.139 |
| viral pneumonia AND influenza AND pneumonia | 0.876 | 0.808 | 0.721 | 0.694 | 0.707 | 0.866 | 0.850 | 5.179 |
| viral pneumonia AND influenza | 0.865 | 0.825 | 0.734 | 0.746 | 0.740 | 0.865 | 0.872 | 5.526 |
| ARDS | 0.846 | 0.796 | 0.762 | 0.563 | 0.648 | 0.912 | 0.807 | 6.398 |
| Influenza | 0.772 | 0.724 | 0.583 | 0.600 | 0.592 | 0.786 | 0.797 | 2.804 |

^a^ACC stands for accuracy. ^b^PPV stands for positive predictive value. ^c^NPV stands for negative predictive value. ^d^PLR stands for positive likelihood ratio.

Table S4. Performance of IMV prediction on COVID-19 patients with different models obtained from the corresponding training cohorts.

| Cohort | AUC | ACC | PPV | Sensitivity | F-score | Specificity | NPV | PLR |
| --- | --- | --- | --- | --- | --- | --- | --- | --- |
|  |  |  |  |  |  |  |  |  |
| Viral pneumonia | 0.826 | 0.948 | 0.423 | 0.289 | 0.344 | 0.981 | 0.966 | 14.918 |
| Viral pneumonia AND pneumonia | 0.778 | 0.927 | 0.138 | 0.105 | 0.119 | 0.968 | 0.957 | 3.255 |
| Viral pneumonia AND influenza AND unspecified pneumonia | 0.760 | 0.936 | 0.250 | 0.184 | 0.212 | 0.973 | 0.960 | 6.781 |
| Viral pneumonia AND influenza AND pneumonia | 0.726 | 0.922 | 0.195 | 0.211 | 0.203 | 0.957 | 0.961 | 4.931 |
| Viral pneumonia AND unspecified pneumonia | 0.705 | 0.946 | 0.286 | 0.105 | 0.154 | 0.987 | 0.957 | 8.137 |
| influenza AND pneumonia AND unspecified pneumonia | 0.700 | 0.914 | 0.136 | 0.158 | 0.146 | 0.951 | 0.958 | 3.212 |
| all | 0.693 | 0.940 | 0.238 | 0.132 | 0.169 | 0.979 | 0.958 | 6.357 |
| Viral pneumonia AND influenza | 0.693 | 0.935 | 0.222 | 0.158 | 0.185 | 0.973 | 0.959 | 5.812 |
| pneumonia AND unspecified pneumonia | 0.679 | 0.910 | 0.170 | 0.237 | 0.198 | 0.943 | 0.962 | 4.161 |
| Viral pneumonia AND pneumonia AND unspecified pneumonia | 0.676 | 0.917 | 0.128 | 0.132 | 0.130 | 0.956 | 0.957 | 2.991 |
| influenza AND unspecified pneumonia | 0.658 | 0.943 | 0.100 | 0.026 | 0.042 | 0.988 | 0.954 | 2.260 |
| pneumonia | 0.652 | 0.917 | 0.128 | 0.132 | 0.130 | 0.956 | 0.957 | 2.991 |
| Unspecified pneumonia | 0.642 | 0.922 | 0.121 | 0.105 | 0.113 | 0.962 | 0.956 | 2.806 |
| ARDS | 0.626 | 0.880 | 0.096 | 0.184 | 0.126 | 0.915 | 0.958 | 2.157 |
| influenza | 0.624 | 0.763 | 0.062 | 0.289 | 0.103 | 0.787 | 0.957 | 1.356 |
| influenza AND pneumonia | 0.615 | 0.878 | 0.082 | 0.158 | 0.108 | 0.913 | 0.957 | 1.822 |

Table S5. Hyperparameter search space for training models.

| Model | Hyperparameter | Description | Search Space |
| --- | --- | --- | --- |
|  |  |  |  |
| AdaBoost | n_estimators | The maximum number of estimators at which boosting is terminated. | [25, 50, 100, 200] |
|  | Algorithm | The discrete boosting algorithm. | [SAMME, SAMME.R] |
| Decision Tree | Criterion | The function to measure the quality of a split. | [gini, entropy] |
|  | max_depth | The maximum depth of the tree. | [None, 5, 6, 7, 8, 9, 10] |
|  | min_samples_split | The minimum number of samples required to split an internal node. | [2, 3, 4] |
| Support-vector machine | C | Regularization parameter. | [0.1, 1, 10, 100, 1000] |
|  | kernel | Specifies the kernel type to be used in the algorithm. | [linear] |
| XGBoost | n_estimators | The function to measure the quality of a split. | [50, 100, 200, 300, 400] |
|  | max_depth | The maximum depth of the tree. | [4, 5, 6, 7, 8] |
|  | learning_rate | Step size shrinkage used in update to prevents overfitting. | [0.01, 0.05, 0.1, 0.2, 0.3] |

Table S6. Hyperparameter search space Selected oversampling method and hyperparameters of the models trained by different cohorts.

| Cohort | Oversampling method | Hyperparameters |
| --- | --- | --- |
|  |  |  |
| viral pneumonia | SVMSMOTE | XGBClassifier(n_estimators=400, learning_rate=0.1, max_depth=4) |
| influenza | SVMSMOTE | XGBClassifier(n_estimators=200, learning_rate=0.05, max_depth=5) |
| ARDS | SVMSMOTE | XGBClassifier(n_estimators=400, learning_rate=0.3, max_depth=8) |
| pneumonia | SVMSMOTE | XGBClassifier(n_estimators=300, learning_rate=0.05, max_depth=8) |
| unspecified pneumonia | SMOTE | XGBClassifier(n_estimators=400, learning_rate=0.1, max_depth=8) |
| viral pneumonia AND influenza | SVMSMOTE | XGBClassifier(n_estimators=100, learning_rate=0.05, max_depth=8) |
| viral pneumonia AND pneumonia | SMOTE | XGBClassifier(n_estimators=400, learning_rate=0.1, max_depth=8) |
| viral pneumonia AND unspecified pneumonia | BorderlineSMOTE | XGBClassifier(n_estimators=400, learning_rate=0.05, max_depth=8) |
| influenza AND pneumonia | BorderlineSMOTE | XGBClassifier(n_estimators=400, learning_rate=0.2, max_depth=8) |
| influenza AND unspecified pneumonia | SVMSMOTE | XGBClassifier(n_estimators=400, learning_rate=0.05, max_depth=8) |
| pneumonia AND unspecified pneumonia | SVMSMOTE | XGBClassifier(n_estimators=300, learning_rate=0.1, max_depth=8) |
| viral pneumonia AND pneumonia AND unspecified pneumonia | SVMSMOTE | XGBClassifier(n_estimators=400, learning_rate=0.05, max_depth=8) |
| influenza AND pneumonia AND unspecified pneumonia | SVMSMOTE | XGBClassifier(n_estimators=300, learning_rate=0.1, max_depth=8) |
| viral pneumonia AND influenza AND pneumonia | SMOTE | XGBClassifier(n_estimators=300, learning_rate=0.1, max_depth=8) |
| viral pneumonia AND influenza AND unspecified pneumonia | SVMSMOTE | XGBClassifier(n_estimators=300, learning_rate=0.1, max_depth=8) |
| all | SVMSMOTE | XGBClassifier(n_estimators=300, learning_rate=0.05, max_depth=8) |

Table S7. Performance of models with best AUC for mortality prediction on different COVID-like cohorts.

| Cohorts | AUC* | ACC | PPV | Sensitivity | F-score | Specificity | NPV | PLR |
| --- | --- | --- | --- | --- | --- | --- | --- | --- |
|  |  |  |  |  |  |  |  |  |
| viral pneumonia AND unspecified pneumonia | 0.748 | 0.771 | 0.624 | 0.215 | 0.320 | 0.957 | 0.785 | 5.000 |
| Viral pneumonia | 0.746 | 0.756 | 0.538 | 0.333 | 0.412 | 0.902 | 0.797 | 3.398 |
| viral pneumonia AND influenza AND unspecified pneumonia | 0.745 | 0.770 | 0.624 | 0.206 | 0.310 | 0.959 | 0.784 | 5.024 |
| viral pneumonia AND influenza | 0.744 | 0.752 | 0.500 | 0.257 | 0.340 | 0.915 | 0.789 | 3.024 |
| influenza | 0.733 | 0.797 | 1.000 | 0.200 | 0.333 | 1.000 | 0.786 | N/A |
| influenza AND unspecified pneumonia | 0.729 | 0.765 | 0.574 | 0.227 | 0.325 | 0.944 | 0.786 | 4.054 |
| all | 0.728 | 0.758 | 0.543 | 0.185 | 0.276 | 0.948 | 0.778 | 3.558 |
| influenza AND pneumonia AND unspecified pneumonia | 0.725 | 0.761 | 0.577 | 0.172 | 0.265 | 0.958 | 0.776 | 4.095 |
| Unspecified pneumonia | 0.718 | 0.760 | 0.545 | 0.248 | 0.341 | 0.931 | 0.788 | 3.594 |
| pneumonia AND unspecified pneumonia | 0.718 | 0.751 | 0.502 | 0.227 | 0.313 | 0.925 | 0.782 | 3.027 |
| viral pneumonia AND pneumonia AND unspecified pneumonia | 0.714 | 0.758 | 0.542 | 0.196 | 0.288 | 0.945 | 0.779 | 3.564 |
| viral pneumonia AND influenza AND pneumonia | 0.703 | 0.752 | 0.508 | 0.239 | 0.325 | 0.923 | 0.784 | 3.104 |
| pneumonia | 0.702 | 0.759 | 0.553 | 0.204 | 0.298 | 0.945 | 0.780 | 3.709 |
| influenza AND pneumonia | 0.697 | 0.738 | 0.457 | 0.274 | 0.342 | 0.892 | 0.787 | 2.537 |
| viral pneumonia AND pneumonia | 0.689 | 0.751 | 0.500 | 0.195 | 0.281 | 0.935 | 0.778 | 3.000 |
| ARDS | 0.679 | 0.731 | 0.578 | 0.308 | 0.402 | 0.907 | 0.759 | 3.312 |

Table S8. Performance of mortality prediction on COVID-19 patients with different models obtained from the corresponding training cohorts.

| Cohorts | AUC | ACC | PPV | Sensitivity | F-score | Specificity | NPV | PLR |
| --- | --- | --- | --- | --- | --- | --- | --- | --- |
|  |  |  |  |  |  |  |  |  |
| all | **0.928** | 0.925 | 0.286 | 0.222 | 0.25 | 0.967 | 0.954 | 6.727 |
| unspecified pneumonia | 0.920 | **0.95** | **0.667** | 0.222 | 0.333 | **0.993** | 0.955 | 31.714 |
| pneumonia AND unspecified pneumonia | 0.919 | 0.912 | 0.333 | **0.556** | **0.417** | 0.933 | **0.972** | 8.299 |
| influenza AND unspecified pneumonia | 0.891 | 0.912 | 0.222 | 0.222 | 0.222 | 0.953 | 0.953 | 4.723 |
| viral pneumonia AND pneumonia AND unspecified pneumonia | 0.869 | 0.906 | 0.250 | 0.333 | 0.286 | 0.94 | 0.959 |  |
| influenza AND pneumonia | 0.840 | 0.906 | 0.125 | 0.111 | 0.118 | 0.953 | 0.947 | 2.362 |
| viral pneumonia AND unspecified pneumonia | 0.815 | 0.918 | 0.250 | 0.429 | 0.316 | 0.973 | 0.942 | 15.889 |
| influenza AND pneumonia AND unspecified pneumonia | 0.810 | 0.881 | 0.222 | 0.444 | 0.296 | 0.907 | 0.965 | 4.774 |
| viral pneumonia AND influenza | 0.806 | 0.925 | 0.286 | 0.222 | 0.25 | 0.967 | 0.954 | 6.727 |
| viral pneumonia AND influenza AND unspecified pneumonia | 0.784 | 0.868 | 0.167 | 0.333 | 0.222 | 0.900 | 0.957 | 3.330 |
| influenza | 0.781 | 0.874 | 0.176 | 0.333 | 0.231 | 0.907 | 0.958 | 3.581 |
| ARDS | 0.749 | 0.931 | 0.25 | 0.111 | 0.154 | 0.98 | 0.948 | 5.550 |
| viral pneumonia | 0.734 | 0.862 | 0.19 | 0.444 | 0.267 | 0.887 | 0.964 | 3.929 |
| viral pneumonia AND influenza AND pneumonia | 0.724 | 0.937 | 0.333 | 0.111 | 0.167 | 0.987 | 0.949 | 8.538 |
| viral pneumonia AND pneumonia | 0.720 | 0.925 | 0.200 | 0.111 | 0.143 | 0.973 | 0.948 | 4.111 |
| pneumonia | 0.687 | 0.931 | 0.25 | 0.111 | 0.154 | 0.980 | 0.948 | 5.550 |

**Figure S1**. Model training process. The figure illustrates the process of how to train a model by training and validation sets. Two steps were involved in model training: 1) Select hyperparameters of models by cross-validation strategy and grid search technique and then obtain the best performance model. 2) retrain the model using all training data with the best hyperparameters obtained in step 1. The validation data were used to monitor the performance of the model to avoid overfitting in training data.


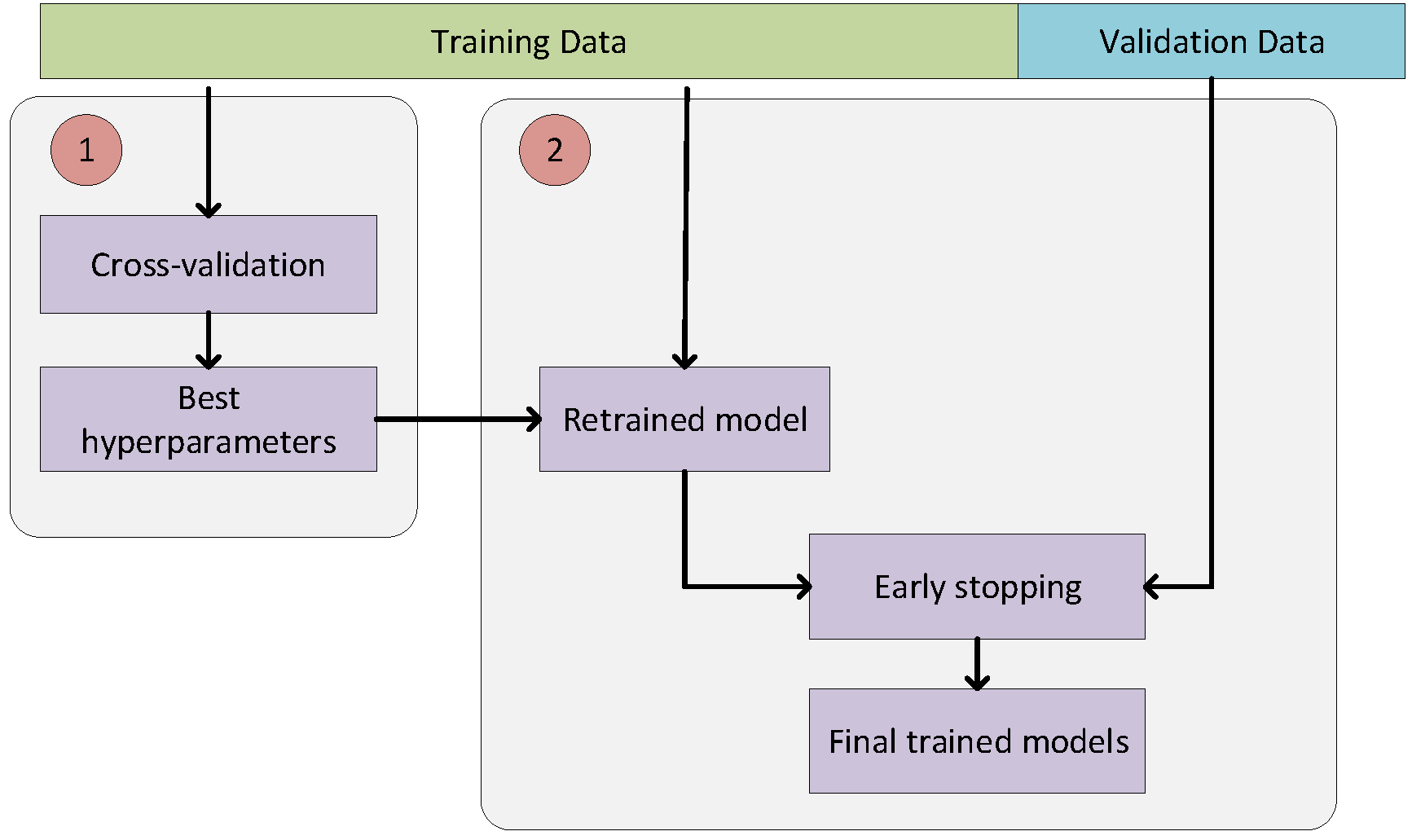

Supplement: Multimedia Appendix 1 [file jmir_v23i2e23026_app1.docx]
